# The Era of Cytotoxic CD4 T Cells

**DOI:** 10.3389/fimmu.2022.867189

**Published:** 2022-04-27

**Authors:** Mara Cenerenti, Margaux Saillard, Pedro Romero, Camilla Jandus

**Affiliations:** ^1^Department of Pathology and Immunology, Faculty of Medicine, University of Geneva, Geneva, Switzerland; ^2^Ludwig Institute for Cancer Research, Lausanne, Switzerland; ^3^Department of Oncology, University of Lausanne, Lausanne, Switzerland

**Keywords:** CD4 T cells, cytotoxic, MHC class II, synapse, immunotherapy

## Abstract

In 1986, Mosmann and Coffman identified 2 functionally distinct subsets of activated CD4 T cells, Th1 and Th2 cells, being key in distinct T cell mediated responses. Over the past three decades, our understanding of CD4 T cell differentiation has expanded and the initial paradigm of a dichotomic CD4 T cell family has been revisited to accommodate a constantly growing number of functionally distinct CD4 T helper and regulatory subpopulations. Of note, CD4 T cells with cytotoxic functions have also been described, initially in viral infections, autoimmune disorders and more recently also in cancer settings. Here, we provide an historical overview on the discovery and characterization of cytotoxic CD4 T cells, followed by a description of their mechanisms of cytotoxicity. We emphasize the relevance of these cells in disease conditions, particularly in cancer, and we provide insights on how to exploit these cells in immunotherapy.

## 1 Historical Overview: The CD4 T-Cell Universe

**Figure 1:**
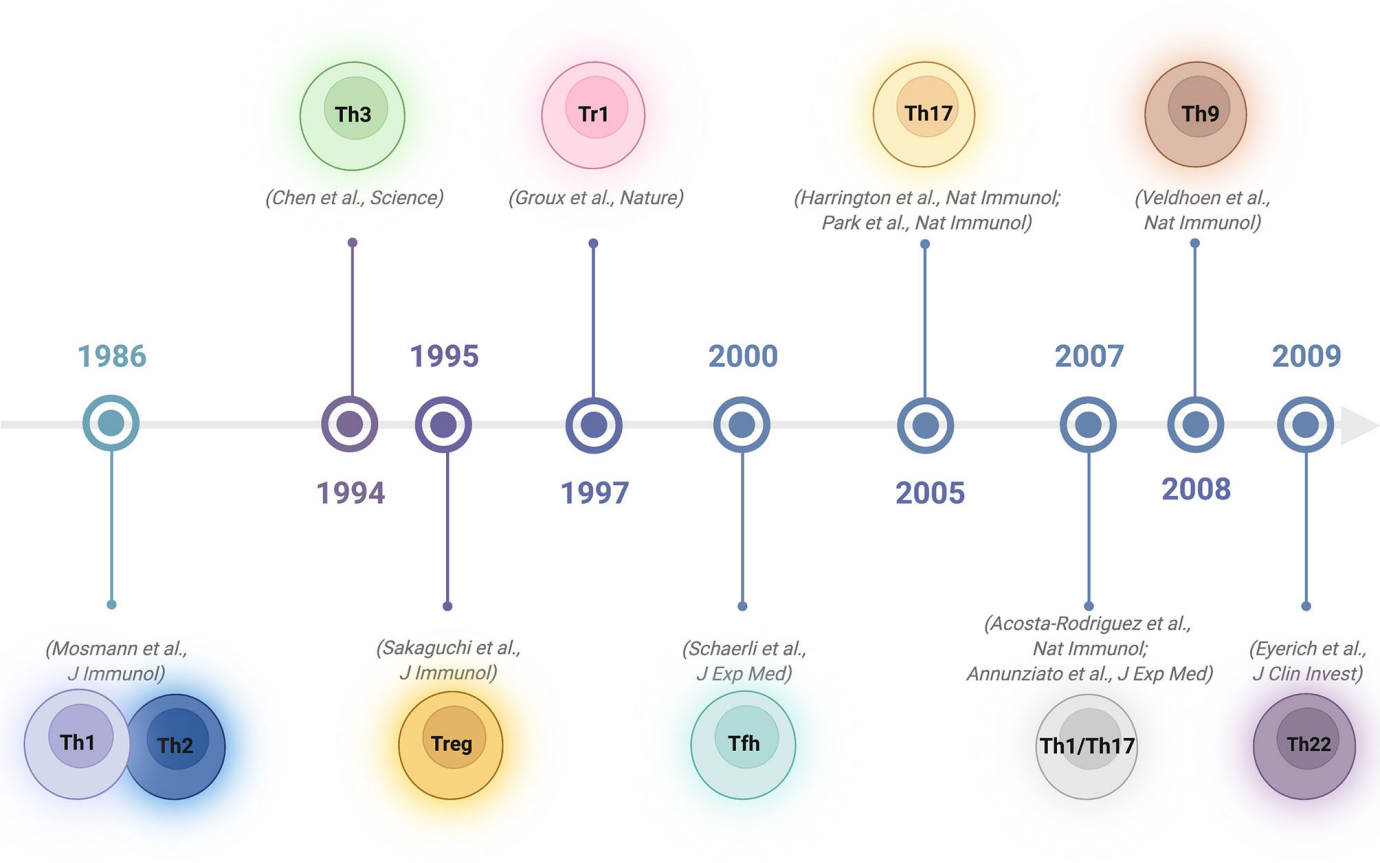
Timeline of discovery of CD4 T helper and regulatory subsets. Created with BioRender.com.

Upon activation, naïve CD4 T cells can differentiate into various specialized subsets characterized by the capacity to produce specific cytokines to promote various types of immune responses (1–3). In 1986, Mosmann and Coffman described 2 types of T cells among CD4^+^ lymphocytes in mice: type 1 T helper (Th1) cells, producing interleukin 2 (IL-2), interferon-γ (IFN-γ), granulocyte-macrophage colony-stimulating factor (GM-CSF), and type 2 T helper (Th2) cells, producing IL-4, IL-5, B-cell-stimulating factor 1 (BSF-1) and mast-cell growth factor (MCGF) (4). The categorization of Th1 and Th2 cells provides a framework for explaining the T-cell immunopathology of many diseases (5). By the beginning of the 1990s, it became apparent that CD4 T-cell clones showing Th1 or Th2 profiles could also be found in tissues or peripheral blood in humans (6, 7). Subsequently, other subsets with different functions have been reported. In 1994, it was shown that oral tolerance regimens induce transforming growth factor β (TGF-β)−producing CD4 T regulatory cells, a subset named Th3 cells (8). In 1995, regulatory T cells constitutively expressing the molecule CD25 were discovered (9), followed in 2003 by the identification of Foxp3 as the master transcription regulator for these cells (10, 11). In 1997, Roncarolo’s group identified a CD4 T-cell subset with low proliferative capacity producing high levels of IL-10, low levels of IL-2 and no IL-4. As these cells suppressed antigen-specific immune responses and downregulated pathological immune responses, they were named T regulatory 1 (Tr1) cells (12). A few years later, CD4 follicular helper T (Tfh) cells were identified as specialized providers of B-cell help necessary for the formation of germinal centres and for the regulation of T-cell-dependent B-cell differentiation into plasma and memory B cells (13). In 2005, a previously unrecognized population of CD4 cells that did not produce the classical Th1/Th2 cytokines, but did produce IL-17, was discovered: so-called proinflammatory Th17 cells (14, 15). Not long after, Th17 cells capable of converting into hybrid Th1/Th17 lymphocytes by combined IFN-γ and IL-12 signalling were observed in specific infectious conditions as a distinct cell subpopulation (16, 17). In 2009, a human proinflammatory Th subset characterized by the secretion of IL-22 and TNF, but not IFN-γ, IL-4 or IL-17, was reported. Since this subset had a profile distinct from those of Th1, Th2 and Th17 cells, this new subset was named Th22 cells (18). Furthermore, an additional subgroup induced by TGF-β and IL-4 and characterized by the production of IL-9 was added to the CD4 T helper family: Th9 cells (19) (**Figure 1**). While the above classification relies on defining CD4 T-cell subsets based exclusively on their dominant secreted cytokine, with the development of new technologies that can screen multiple markers, integrins or chemokines at the single-cell level, alternative categorizations have been proposed. Specifically, instead of focusing on the type of T helper cell, which might be plastic and evolve over time, viewing the system from the perspective of the target cell or the type of immune response induced has been suggested (20, 21). In this way, complex and integrated helper functions rather than helper phenotypes would be prioritized.

While the discussion on this “helper cell” nomenclature matter has just initiated, evidence on the functional relevance of CD4 T cells with cytolytic activity, an attribute that was believed for decades to be restricted to CD8 T cells, is increasing. Initially, CD4 cytotoxic T lymphocytes (CD4 CTLs) were considered a potential artefact of *in vitro*-generated T cells. This idea was challenged by reports providing evidence that some antigen-specific CD4 T cells *in vivo* possess direct MHC class II-restricted cytotoxic activity, as initially described in the 1970-1980s both in humans (22, 23) and mice (24, 25). Since then, the number of conditions showing the presence of CD4 CTLs in both species has grown steadily. However, open questions remain regarding their exact phenotype, their mechanism(s) of action, their potential ability to transition towards/from the CD4 helper lineages and their prospective usefulness as therapeutic agents. We will discuss these aspects in the following sections.

## 2 CD4 CTLs in Pathologic Conditions

Under physiological conditions, CD4 CTLs represent a small percentage of circulating CD4 T cells, primarily identified within highly differentiated effector cells. Single-cell transcriptomic analyses combined with T-cell receptor (TCR) sequencing showed that putative CD4 CTL precursors express high levels of the IL-7 receptor and undergo significant clonal expansion during pathological processes (26). Of note, an unexpected strong expansion of these cells was observed in supercentenarians, in whom CD4 CTLs represented up to 25% of total CD4 T cells. This accumulation appears, at least in part, to be the consequence of clonal expansion following repeated viral exposure, suggesting that CD4 CTLs are essential for achieving longevity because they successfully protect against infections and diseases (27). Similarly, in a previous work, CD4 CTLs, characterized by the expression of NKG2D, granzyme B and perforin, were shown to be significantly enriched in elderly people compared to young adults (28). This increase in cytotoxic, highly differentiated T cells in aged individuals might represent the accumulation of senescent immune cells driven by multiple persistent stimuli such as cumulative viral challenges and age-dependent emergence of somatic cells with genetic abnormalities generating neo-antigens.

### 2.1 Infectious Diseases

Initial studies reported the *in vivo* presence of CD4 CTLs in various viral infections. Specifically, CD4 CTLs have been observed among human peripheral blood mononuclear cells (PBMCs) in chronic viral infections, such as infections with human cytomegalovirus (CMV) (29–31), human immunodeficiency virus 1 (HIV-1) (32, 33) and hepatitis viruses (HBV, HCV, and HDV) (34). CD4 CTLs have also been described in mice infected with chronic viruses, including lymphocytic choriomeningitis virus (LCMV) (35, 36) and gamma-herpes virus (37). In mice affected by murine cytomegalovirus (MCMV) infection, virus-specific CD4 T cells with cytolytic capacity mediated vaccine protection *via* multiple effector mechanisms *in vivo* (38). CD4 T cells with cytotoxic capacity were also found in Epstein–Barr virus (EBV)-infected patients and mice (39). In patients, the virus induced the expansion of antigen-specific CD4 CTLs (40), and these cells were able to recognize and eliminate infected B cells (41). Polyfunctional and CD4 CTLs were reported in human herpes virus (HHV)-6B-infected individuals and linked to long-term disease control (42). In line with these protective roles, CD4 CTLs were also detected in patients affected by Dengue, a mosquito-borne viral disease that has rapidly spread in recent years. Dengue virus (DENV)-specific CD4 T cells had direct *ex vivo* cytolytic activity and were enriched in patients carrying HLA histocompatibility alleles associated with disease protection, suggesting that DENV-specific CD4 CTLs may directly contribute to the control of severe dengue pathology *in vivo* (43). Furthermore, CD4 T cells with killing capacity expand in response to influenza virus infection (44–46), where they show a phenotype typical of Th1 effector cells but express granzyme B and perforin, contributing to protection against influenza A virus (IAV) infection both in mice and humans (47). Recently, CD4 CTLs have been identified in SARS-CoV-2-infected patients (48). Specifically, increased proportions of SARS-CoV-2-reactive CD4 CTLs and a unique population of CD4 follicular helper T cells enriched in cytotoxicity-associated transcripts were observed in hospitalized patients with impaired humoral responses, suggesting that these cells might be involved in the loss of germinal centre B cells observed in SARS-CoV-2 patients who succumb to the disease (49). A similar cytotoxic Tfh population was recently described in children with recurrent tonsillitis (50). Finally, CD4 CTLs can also confer protection against malaria infection both in mice (51) and in humans (52) by producing IFN-γ. New studies relying on mass cytometry, multidimensional flow cytometry, single-cell transcriptomics analyses and cellular indexing of transcriptomes and epitopes by sequencing (CITE-seq) analyses at the single cell level are expected to shed light on the relative frequencies of CD4 CTLs compared to classical cytotoxic lymphocytes and on key phenotypic markers to specifically identify these cells.

### 2.2 Autoimmune Diseases

CD4 CTLs have also been detected in autoimmune diseases (53, 54). Multiple sclerosis (MS) is the leading cause of chronic neurological disability in young adults. Different groups have shown that in an animal model of MS, CD4 CTLs drive progression of the disease, providing a link between the presence of these cells and disease severity and significant implications of these cells as therapeutic targets (55, 56). Primary Sjogren’s syndrome (SS) is one of the most common autoimmune diseases, and its pathogenesis remains poorly understood. Expansion of CD4 CTLs was identified in SS patients by single-cell RNA sequencing, and these cells might be involved in the pathogenesis of the disease (57). In addition, in ulcerative colitis (UC), CD29^+^ CD4 T cells were described as effectors leading to persistent inflammation and were involved in the repeated inflammation bouts observed in this disease (58). In another severe autoimmune disorder, systemic lupus erythematosus (SLE), CD4 T cells expressing natural killer group 2D (NKG2D) are expanded NKG2DL^+^ Treg cells that remove crucial immune-suppressive cells (59). CD4 CD28^-^ T cells producing IFN-γ and perforin were reported *ex vivo* in samples taken from patients with rheumatoid arthritis (60) and ankylosing spondylitis (61).

### 2.3 Cancer

While T-cell studies in cancer have mainly focused on CD8 T cells, given their direct tumoricidal activities and the lack of MHC class II expression in many cancer types, recent data argue for a crucial contribution of CD4 T cells to tumour immunity (62). The protumour *vs*. antitumour roles of helper and regulatory CD4 T-cell subsets have been extensively studied in different tumour types. In contrast, the existence and function of CD4 CTLs in cancer remain unclear. Seminal preclinical studies by Allison’s group demonstrated that CD4 CTLs can directly kill tumour cells and eradicate established tumours in an MHC class II-dependent manner (63). In line with these observations, it was reported that tumour-reactive CD4 T cells with tumoricidal activities expand *in vivo* and eradicate established melanoma after the transfer of naïve CD4 T cells into lymphopenic hosts (64). Subsequent studies in patients showed the presence of expanded CD4 CTLs in several tumour types, such as lung cancer (65), colorectal cancer (66), hepatocellular carcinoma (67, 68), breast cancer (69, 70), head and neck cancer (71), osteosarcoma (72) and malignant melanoma (73), as assessed by deep single-cell RNA sequencing analyses of tumour-infiltrating lymphocytes. Whether these cells exacerbate, or counter tumour progression or metastasis formation remains to be fully elucidated and might depend on the tumour type and/or stage. Recently, CD4 T cells displaying a cytotoxic gene signature were reported in children with high-risk neuroblastoma and were associated with a putative protective effect that declined over time due to the progressive formation of an immunosuppressive tumour microenvironment (74). CD4 CTLs were also observed in B-cell chronic lymphocytic leukaemia (B-CLL), where they were able to kill autologous B-CLL cells *ex vivo* in a perforin-mediated mechanism (75, 76). In Burkitt’s lymphoma (BL), CD4 CTLs recognize an epitope of EBV, providing a novel mechanism for immune targeting of EBV-positive malignancies, as EBV-associated malignancies often escape class I-restricted immune recognition (76). Moreover, given that malignancies of B-cell origin express high levels of MHC class II, direct cytotoxicity by CD4 T cells might be the dominant mechanism for their elimination. Single-cell transcriptomic analyses in bladder cancer patients have recently identified multiple states of intratumoral CD4 CTLs. Of note, in a cohort of 244 metastatic bladder cancer patients treated with anti-PD-L1 therapy, a gene signature of CD4 CTLs was predictive of clinical response, arguing for a contribution of CD4 CTLs to the therapeutic efficacy of immune checkpoint (IC) blockade (77). However, whether and how CD4 CTLs can eliminate bladder cancer cells *in vivo* remain to be determined. The observations in bladder cancer are supported by previous preclinical work in murine melanoma. Transfer of a small number of CD4 T cells into lymphopenic mice, in combination with CTLA-4 blockade (63) or CD137 agonist immunotherapy (78), resulted in potent rejection of large vascularized tumours, independent of other immune cells and in an MHCII-restricted manner. Overall, these findings emphasize the possibility of exploiting the functions of CD4 CTLs in cancer immunotherapy, as discussed in more detail in Section 5 of this Review.

## 3 Phenotype and Killing Mechanisms of CD4 CTLs

### 3.1 Phenotype of CD4 CTLs

**Figure 2:**
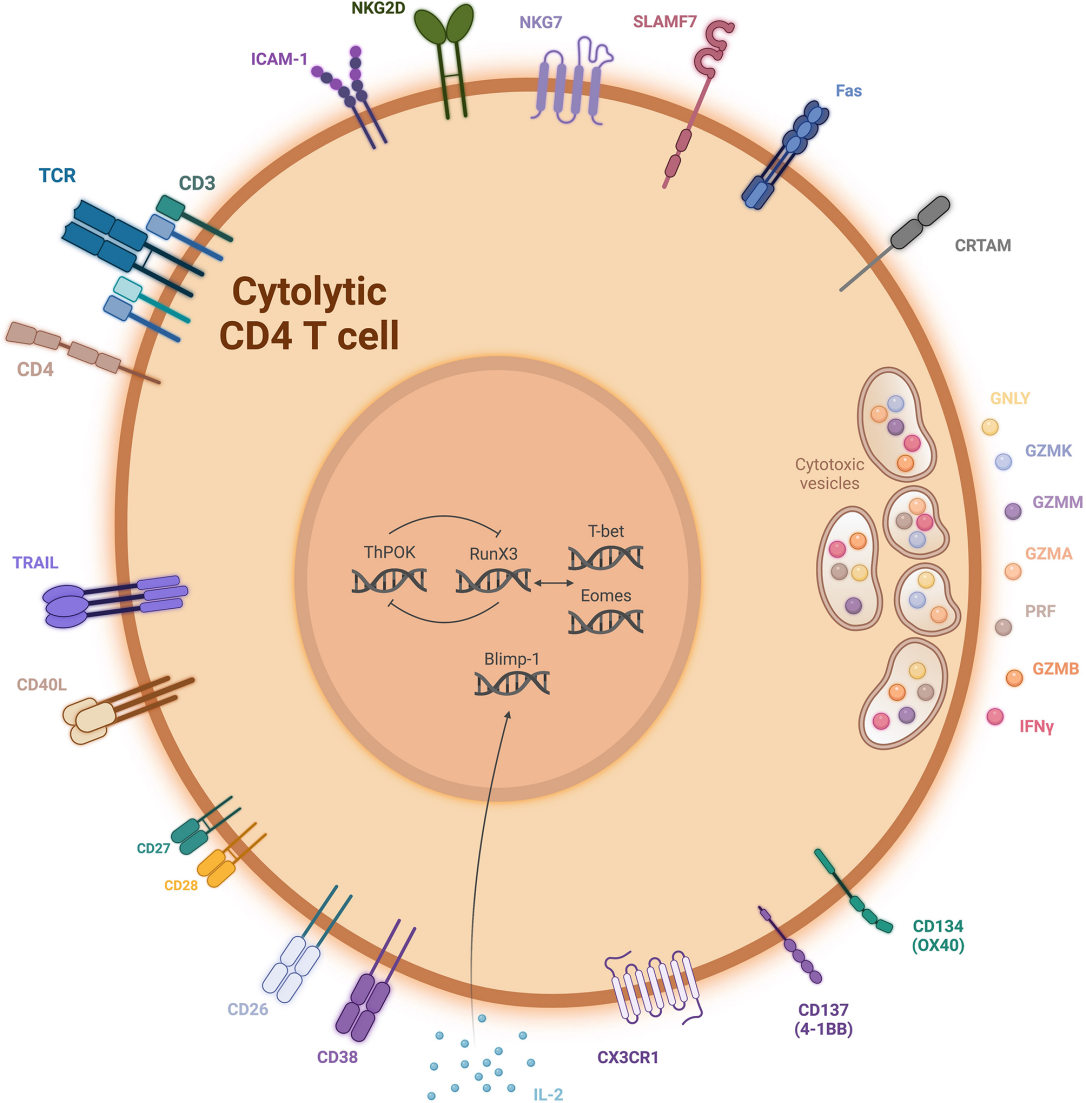
Schematic representation of the cell surface phenotype and some of the transcriptional mechansims that might contribute to CD4 CTL activation. Created with BioRender.com.

Although CD4 T cells with cytotoxic functions have been known for decades, it remains difficult to define a set of surface markers or transcription factors to differentiate CD4 CTLs from helper CD4 T-cell subsets (32). Here, we provide an overview of markers and transcription factors that have been implicated in defining CD4 CTLs (**Figure 2**).

CD4 CTLs are mainly found within effector/effector memory, antigen-experienced, highly differentiated cells that have downregulated costimulatory receptors such as CD27 and CD28 (32, 61). Nevertheless, their differentiation pathways remain largely unknown. Transcription factors from the T-box family (e.g., T-bet (T-box expressed in T cells), Eomes (eomesodermin) and Runx3 (runt-related transcription factor 3) are known to cooperate to establish cytotoxic programs in CD8 T cells (79). In parallel, it has been suggested that both T-bet and Eomes are upstream inducers of the cytolytic capacity of CD4 T cells (80–82). In line with this hypothesis, Eomes is required for granzyme B expression by cytotoxic CD4 Th1 cells after dual CD134 and CD137 costimulation (83). Furthermore, ThPOK (T-helper-inducing POZ/Krueppel-like factor) is known to suppress Runx3 and to maintain CD4 T-cell lineage specification during thymic development (84). In mature T cells, sustained ThPOK expression limits the acquisition of Runx3-dependent cytotoxic functions in CD4 T cells (85). The balance of transcription factors expressed in peripheral CD4 T cells also influences the plasticity between helper and cytotoxic phenotypes, with persistent expression of ThPOK allowing the maintenance of the helper T-cell lineage gene expression program. Conversely, downregulation of ThPOK expression drives the conversion of mature CD4 T cells into MHC class II-restricted cytotoxic T lymphocytes (86). Finally, it has recently been shown that depletion of Treg cells induces a surplus of IL-2 in the tumour microenvironment in mice. In response to IL-2, the transcription factor Blimp-1 (B lymphocyte-induced maturation protein-1) drives granzyme B production and supports the acquisition of cytotoxic activity by T helper cells (87). Interestingly, the homologue of Blimp-1 in T cells, also called Hobit, which is mostly associated with tissue residency (88, 89) and maintenance of effector functions in CD8 T cells, appears to be equally linked to cytotoxic potential in CD4 T cells after primary hCMV infection (90).

In addition to putative master transcription factors, a highly variable panel of surface biomarkers has been proposed to define CD4 CTLs. For instance, among naïve and memory CD4 T cells, a small cell fraction expresses MHC class I-restricted T-cell-associated molecule (CRTAM) upon activation. CRTAM acquisition occurs in association with a heightened cytolytic capacity linked to the expression of cytolytic-related genes such as Eomes, IFN-γ, granzyme B and perforin (91). In line with these findings, in mouse models of viral infection, an increase in CRTAM-positive CD4 T cells was observed, but its expression was only transient upon TCR stimulation, making this molecule difficult to use as a specific CTL marker *in vivo*. CRTAM was originally described as an early activation marker of NK and CD8 T cells and plays a role in the regulation of CTL and NK-cell function (92). In malaria-infected individuals, CD38-positive CD4 T-cell expansion correlates with a significant decrease in the parasite burden in the blood, demonstrating a potential cytolytic function of these cells. CD38 is a glycoprotein with ectoenzymatic functions, and CD38^+^ CD4 T cells can also be identified in healthy donors, but only at lower frequencies (52). Furthermore, NKG2D^+^ CD4 T cells express cytotoxic factors such as perforin, granzyme B and FasL and have been shown to efficiently kill NKG2DL^+^ Treg cells (59). NKG2D is a key activating receptor expressed in NK cells (93), arguing for putative TCR-MHC-independent cytotoxic activity of NKG2D-expressing CD4 T cells. Experiments using MHC class II-blocking antibodies would help to unravel this mechanistic aspect. We recently reported that another NK-cell-associated molecule, signalling lymphocyte activation molecule family member 7 (SLAMF7), is enriched in CD4 CTLs in cancer patients. Its agonistic triggering can increase MHC class II-dependent target cell killing, at least *in vitro* (73). A humanized anti-SLAMF7 antibody (elotuzumab) has proven successful in the treatment of multiple myeloma patients. Whether its therapeutic efficacy also depends on the triggering of CD4 CTLs warrants investigation in the near future (94). Natural killer cell granule protein 7 (NKG7), expressed by NK cells, is also specifically enriched in CD4 TEMRA cells, concomitantly with transcripts for granzyme B, perforin and granulysin in cells displaying high cytotoxic potential (26). In addition, DENV-specific CD4 T cells upregulate the fractalkine receptor CX3CR1, previously described in NK cells and cytotoxic effectors (95), which correlates with cytotoxic capacity and Eomes, granzyme and perforin expression (43). Collectively, the fact that several prototypic NK-cell receptors are overexpressed by CD4 CTLs suggests that they might be indispensable for fulfilling the full cytotoxic potential of these cells. In HIV, CD107a^+^IFN-γ^+^ double-positive CD4 T cells share a transcriptional profile, including the expression of granzymes A and B and perforin, and exhibit killing activity similar to that of HIV-specific cytolytic CD8 T cells (96), suggesting that this marker combination might appropriately define CD4 CTLs, at least in the case of HIV infection. CD26, a widely expressed glycoprotein with dipeptidyl peptidase IV (DPPIV) activity, was recently proposed as a new marker for CD4 CTLs. Indeed, CD26^high^ CD4 T cells elicit potent immunity against solid tumours (97). Finally, CD4 CTLs with the ability to kill autologous B cells can be induced by a TLR4 agonist adjuvant, resulting in the induction of CD40L and engagement of CD40 on target cells (98).

### 3.2 Mechanisms of Killing by CD4 CTLs

In terms of cytotoxic effector molecules, in addition to the release of lytic granules containing granzymes and the expression of perforin, other mechanisms might be involved in CD4 CTL cytotoxicity.

#### 3.2.1 Granule-Dependent Cytotoxicity

Transcriptomic data confirmed by protein quantification revealed the presence of the antimicrobial peptide granulysin, and different granzyme types, including granzymes K and M, were significantly enriched in CD4 CTLs (73, 77). In addition, CD4 CTL, CD8 CTL and NK populations share similar expression of genes associated with granule-dependent cytotoxicity, including perforin, granulysin, granzymes A and B, although granzymes K, H and M levels are lower in CD4 than in CD8 CTLs (99, 100). Granulysin is a cationic protein with bactericidal activity. Its presence in CD4 CTLs suggests that these cells might be helpful in the response to some bacterial infections inefficiently cleared by conventional cytotoxic cells. Regarding granzymes, a recent report on CLL patients with durable responses to CD19-specific CAR T-cell therapy showed decade-long persistence of a highly activated CD4 T-cell population displaying upregulation of granzyme K and its closest homologue, granzyme A. These 2 genes were among the top 4 genes mostly upregulated in the CAR CD4 T cells. In contrast, granzyme B and M were highly expressed in persisting CAR CD8 T cells (101). Overall, CD4 T-cell cytotoxicity seems to be dependent, at least in part, on granule secretion but might rely on serine proteases with both cytotoxic and noncytotoxic functions (such as granzyme K) other than those employed by CD8 T cells.

#### 3.2.2 Death Receptor-Dependent Cytotoxicity

In addition, the involvement of the Fas-FasL pathway has been reported in some studies, while Fas-FasL pathway-independent cytotoxicity has been observed in other settings (73, 102). Previously, it was shown that the use of monoclonal antibodies against Fas did not inhibit the *in vitro* killing of melanoma cells by CD4 CTLs (103). Another death receptor, TNF-related apoptosis-inducing ligand (TRAIL), can induce cancer cell death *via* apoptosis and is considered a cytotoxic marker (104). In melanoma, the TRAIL-TRAIL receptor axis can mediate cytotoxic activity by CD4 T cells against tumour cells (105). In contrast, in CD8 T cells and NK cells, Fas-FasL and TRAIL-TRAIL interactions are part of the major mechanism implicated in the destruction of target cells (106, 107). Overall, many unresolved questions remain in terms of differentiation pathways, markers, and cytotoxic mechanisms of CD4 CTLs. With the development of high-resolution microscopy technologies [with their respective advantages and disadvantages, as recently reviewed by others (108)], in-depth analysis of the immunological synapse between T cells and their targets might help clarify molecular usage, kinetics and mechanistic differences between helper and CD4 CTLs, and the features that endow the latter with the “licence to kill”.

## 4 The Immune Synapse Formed by CD4 CTLs

CD4 T-cell activation depends on interactions between the T-cell receptor (TCR) and its cognate peptide presented by an MHC class II molecule (pMHCII) (109). Several parameters have been found to be responsible for the acquisition of a helper versus a cytotoxic phenotype. The strength of the TCR affinity, the antigen dose (110), and the cytokine environment (110, 111) all contribute to the acquisition of cytotoxic functions, although these parameters are much less well characterized in CD4 CTLs than in cytolytic CD8 T cells (112). Interestingly, using a novel real-time single-cell nanochip, we recently reported that the killing kinetics of human tumour-specific CD4 T cells are delayed compared to those of CD8 T cells, suggesting that CD4 CTLs might rely on a distinct killing mechanism and/or spatiotemporal localization of TCR-pMHC interactions to acquire cytotoxic functions compared to conventional cytolytic lymphocytes (73). Productive T-cell activation requires the formation of the so-called immunological synapse (IS), where the TCR, MHC molecules loaded with an antigenic peptide and costimulatory molecules reorganize and lead to T-cell activation. The canonical view of the synapse refers to the generation of a “bull’s eye structure”, the supramolecular activation cluster (SMAC) (113), consisting of a central TCR-MHC cluster (central SMAC, cSMAC) surrounded by a ring of LFA-1/ICAM-1 adhesion molecules (peripheral SMAC, pSMAC) and a more distal ring where F-actin is concentrated (distal SMAC, dSMAC). The cSMAC can be divided into two components: the endo-cSMAC, in which TCR and CD28 continue to signal, and the exo-cSMAC, composed of TCR-enriched extracellular vesicles (114). Other molecules, such as protein kinase Cθ, are present in the pSMAC, as is CD45 in the dSMAC. CTLs function was reported to be independent of actin or plus-end microtubule motors (115). These cells use a novel mechanism controlled by movement of the centrosome to deliver lethal lytic granules to the target (115). Specifically, the centrosome moves to and contacts the plasma membrane at the cSMAC of the IS. Therefore, once the IS is formed, in CTLs, the secretory granules relocalize to the microtubule-organizing centre (MTOC) and ultimately polarize towards the IS, where actin depletion plays a critical role in regulating secretion (116). While very important for efficient delivery of the cytotoxic hit through the release of perforin and granzyme in the synaptic cleft, MTOC relocalization has been shown to be neither absolutely indispensable (117) nor necessary for lytic granule release (118). In parallel, in FasL-dependent cytotoxic cells, relocalization of the FasL molecule from the lysosome to the cell membrane occurs to trigger apoptosis of Fas-expressing targets (119). The overall duration of these synapses in cytotoxic CD8 T cells is very short, lasting only a few minutes, thus enabling repeated successive encounters with several target cells that can be serially killed. In comparison, the IS of conventional helper CD4 T cells is a much more stable structure that persists hours for optimal and continuous cytokine secretion (120). In this case, MTOC-containing cytokine-loaded granules traffic much slower to the IS, although consecutive formation of ISs with different targets can also occur for helper CD4 T cells. Finally, studies in CD4 CTLs showed that they form different ISs than either cytolytic CD8 T cells or helper CD4 T cells. Unstable cytolytic synapses were observed, with the delivery of the granules mostly in the pSMAC as opposed to in the cSMAC, as seen in the case of cytotoxic CD8 T cell ISs. In line with these findings, activated src kinases, reflecting proximal TCR-mediated signalling, were observed in both the cSMAC and the pSMAC of CD8 CTLs, while they were observed only in the pSMAC of CD4 CTLs. Of note, it is important to highlight that it is possible to modify IS stability. Treatment of CD4 CTLs with a protein kinase Cθ` inhibitor, which controls the pSMAC ring, increases synapse stability and the effectiveness of target cell lysis (121).

CTL synapses can have a polarized or nonpolarized pattern of degranulation; this latter case has been described in NK cells, where granule movement is uncoupled from MTOC polarization during synapse assembly (122). Moreover, while signalling at the pSMAC is not able to promote CTL polarization, totally depending on TCR engagement, in NK cells, LFA-1 signalling is sufficient to promote MTOC and granule polarization at the immune synapse (123). Other studies on NK cells have further confirmed that Wiskott-Aldrich syndrome protein (WASP) and WASP-interacting protein regulate polarization towards synapses (124). Notably, even though actin dynamics are also important for the formation of the NK immune synapse, a crucial difference exists between the synapses of NK cells and CTLs in the cortical cytoskeleton distribution. In contrast to the case in CTLs, in NK cells, lytic granule secretion occurs through a dense F-actin meshwork containing granule-sized clearances (125, 126). Additionally, NK-cell granules are constitutively associated with the motor myosin IIA, which promotes their interaction with the F-actin-rich cell cortex at the synaptic membrane and assists their final transit towards the synaptic cleft (127).

Little is known about the signalling pathways that trigger granule trafficking along the microtubules and determine the directionality of their transport, although a few signalling parameters have emerged as important regulatory factors, such as the signal strength. TCR triggering with low-affinity ligands leads to impairment of lytic granule polarization towards the MTOC (128), whereas only high-avidity interactions give rise to granule recruitment to the polarized centrosome at the synapse. Moreover, increased signal strength leads to an increased proportion of CTLs, where TCR strength modulates the rate but not the organization of effector CTL responses (129). Consistent with these findings, a study in CD4 T cells also found that stronger TCR signals resulted in decreased levels of PIP2 (130).

Overall, delayed and less effective cytolytic responses were observed in side-by-side studies of CD8 and CD4 CTLs, as was a lower propensity to kill a greater number of target cells within a limited time (131). Nevertheless, these cells are emerging as crucial cytolytic players in the context of *in vivo* MHC class I loss, as frequently seen in cancer. MHC class I downregulation or mutations in genes associated with MHC class I expression have been reported as the dominant mechanism of primary or secondary therapy resistance (132–134). The cytokine secretion capacity of CD4 CTLs linked with their cytotoxic functions might compensate for the loss of direct CD8-mediated killing in patients with defects in antigen presentation by MHC class I molecules.

## 5 CD4 CTLs in Immunotherapy

Targeting CD4 T cells in immunotherapy is receiving increasing attention owing to their pleiotropic antitumor roles, such as the ability to induce senescence of tumour cells (135, 136), to trigger the generation of tumoricidal macrophages (137, 138), to drive cytokine-dependent destruction of endothelial cells (139), and to help CD8 T cells, and more recently, they have also been recognized for their direct cytotoxic activity against tumour cells. In this section, we will discuss current evidence for CD4 CTL targeting and triggering in cancer immunotherapy.

### 5.1 Adoptive T-Cell Transfer

Preclinical models showing successful tumour rejection after the transfer of a small number of CD4 T cells into preconditioned tumour-bearing animals provided initial evidence for the clinical potential of CD4 CTL-based adoptive cell transfer (ACT) therapy (63, 78). In line with these observations, naïve tumour/self-specific CD4 T cells naturally differentiated into Th1/cytotoxic T cells *in vivo* and were sufficient to induce regression of murine melanoma (64). These cells expressed Tbet, IFN­γ, CXCR3, granzyme B, perforin and LAMP­1. Furthermore, it was recently shown that human CD4 CD26^high^ T cells engineered to express a mesothelin-chimeric antigen receptor (CAR) elicit stronger immunity against large established mesothelioma after adoptive transfer in NSG mice than other Th CD4 subsets engineered with the same CAR (97). In humans, a single infusion of clonal NY-ESO-1-specific CD4 T cells in a metastatic melanoma patient resulted in complete resolution of pulmonary and nodal disease 2 months after ACT, suggesting that CD4 T cells alone were sufficient to trigger tumour elimination (140). In another case report study, tumour regression was induced in a metastatic epithelial cancer patient by ACT of endogenous tumour-infiltrating CD4 T cells recognizing a mutated erbb2 protein (141). When the disease progressed, the patient was retreated with mutation-reactive CD4 T cells and experienced tumour regression again. Rosenberg’s group evaluated the safety and efficacy of ACT using TCR-engineered CD4 T cells that expressed an HLA-DP4-restricted TCR targeting the cancer-testis antigen MAGE-A3. This regimen showed for the first time evidence that objective tumour regression can be mediated by engineered MAGE-A3–specific CD4 T cells in a variety of cancer types (142). More recently, Inderberg’s group isolated a human telomerase reverse transcriptase (hTERT)-specific TCR was identified in a CD4 T-cell clone from a vaccinated pancreatic cancer patient that, when expressed in primary CD4 and CD8 T cells, conveyed potent killing efficacy and reduced tumour growth, leading to improved survival in a xenograft mouse model (143). Current efforts should focus on refining the criteria to select optimal CD4 CTLs in order for CD4 T cells to be implemented in ACT-based therapies and to achieve ultimate clinical success. In this regard, it is crucial to understand the regulation of CD4 CTL induction and the possibility of preferentially triggering CD4 CTLs *in vitro* for ACT. The use of histone deacetylase inhibitors (HDACis) resulted in upregulated cytotoxic-related genes in CD4 T cells, arguing for epigenetic control of CD4 T-cell helper versus cytotoxic phenotypes (144). Antigen dose also influences CD4 T-cell cytolytic activity: a low concentration of peptide induces more potent cytolytic activity than relatively high doses, particularly *via* IL-2 (110). Moreover, costimulation with CD134 (OX40) and CD137 (4-1BB) maximizes clonal expansion and imprints a cytotoxic phenotype on CD4 T cells (83). It has been reported that IL-12 can increase the granzyme expression and cytotoxicity of CD8 T cells (145), but this remains to be tested in CD4 T cells. In addition, we showed that exposure to IL-12 increased SLAMF7 expression in CD4 T cells (73). Finally, it is noteworthy that TCR signal strength affects the differentiation of effector cells and T-cell polarization, as it controls downstream cytokine receptor expression (146). These data suggest that it is possible that CD4 CTL differentiation is similarly regulated.

In addition to the transfer of natural or gene-engineered T cells, chimeric antigen receptor (CAR)-T-cell ACT is also emerging as a powerful immunotherapy, mainly for haematologic malignancies. Whether high proportions of CD4 T cells in the infusion product lead to superior results has only started to be determined. In leukaemia, *in vivo* generation of CD19-CAR T cells selectively in CD4 T cells by using a CD4-targeted lentiviral vector led to the reduction or even complete elimination of CD19-positive cells (147). In addition, in a tumour mouse model, these cells exhibited superior tumour cell killing and faster kinetics than CD8-targeted lentiviral vector counterparts (148). Interestingly, in 2010, two patients with CLL were infused with CD19-specific CAR T cells and responded with complete remission. A recent analysis of the CAR T-cell populations in the 10-year follow-up of these patients showed the persistence of highly activated CD4 T-cell populations with cytotoxic characteristics, such as high granzyme K and A expression, which appear to be critical for long-term tumour control, as opposed to CD8 CAR T cells, which are key in the initial response phase (101). In addition, in glioblastoma, CD4 CAR T cells were identified as a highly potent and clinically important T-cell subset for therapy (149).

### 5.2 Immune Checkpoint Blockade

The putative involvement of CD4 CTLs in clinical responses to immune checkpoint blockade (ICB) stems primarily from correlative or *in vitro* studies. Baseline expression of *in situ* MHC class II, but not MHC class I, by tumour cells was reported to be sufficient to segregate responders from non-responders treated with an anti-PD1 antibody (150). This finding suggests a potential direct contribution of CD4 CTLs to ICB clinical efficacy. In line with these data, in bladder cancer, an intratumoral cytotoxic CD4 gene signature was predictive of the response to anti-PD-L1 therapy (77). In 4 melanoma patients treated with anti-CTLA4, tumour-specific CD4 T-cell lines established from samples collected post-ipilimumab treatment showed superior *in vitro* lysis of NY-ESO-1^+^-expressing tumour cell lines compared to pre-treatment CD4 T cells (82), suggesting ICB induction of a CD4 CTL phenotype. These results are supported by preclinical work combining ACT and ICB in tumour mouse models (63). Similarly, *in vitro* OX40 engagement by three patient-derived tumour-specific CD4 T-cell lines exhibited heightened cytolytic effects against melanoma cell lines, arguing for *in vivo* tumoricidal capacity, as observed in the preclinical evaluation (81). How the targeting of distinct inhibitory or activating receptors impacts pre-existing CD4 CTLs and/or induces them *de novo* remains to be fully elucidated and will be highly relevant for patient stratification and immune treatment choice. Furthermore, in addition to correlative studies, direct side-by-side comparisons of CD4 CTLs and CD8 T cells in appropriate NSG or humanized mouse models will be necessary to prove the clinical relevance of these cells, alone or in combination with CD8 T-cell targeting.

### 5.3 Vaccination

The inclusion of CD4 T-cell targeting in vaccination protocols has recently led to superior, integrated CD4 and CD8 T-cell responses in cancer patients. While T helper and T regulatory cell responses have been extensively characterized in trials consisting of several vaccine regimens, studies evaluating CD4 CTL expansion/induction and their clinical relevance upon therapeutic vaccination in cancer remain scant. The first vaccine that induced a CD4 CTL response was published in the 1990s in human immunodeficiency virus type 1 (HIV)^+^ individuals. In that study, patients were vaccinated with recombinant envelope glycoprotein gp160. Cytotoxic activity was observed and was found to not be mediated by classic CD8 CTLs but rather by cells of the CD4 T-cell lineage that were able to lyse targets expressing HIV-1 (151). Around the same time, a case study showed that a vaccination consisting of a mutated p21ras peptide-induced CD4 CTL antigen-specific T cells that were able to recognize pancreatic-adenocarcinoma cells achieved a successful outcome (152). In a recent trial based on long synthetic peptides targeting up to 20 neoantigens per patient, both CD4 and CD8 neoantigen-specific T-cell responses were generated. Gene expression profile analyses in individual neoantigen-reactive CD4 T cells showed an upregulation of genes related to cytotoxicity, such as granzyme A and granulysin (153). Peptide cancer vaccines also stimulated CD4 T cells with cytotoxic capacity in prostate cancer patients after an AE37 vaccine, a HER2 hybrid polypeptide. These cells share a Th1 cytokine profile, which contributes to strengthening effector antitumor functions (154). Additionally, another trial also demonstrated the generation of antigen-specific CD4 T cells cytotoxic against hTERT^+^ cells. The presence of these cells in combination with CD8 T cells elicited an important response essential for tumour regression and the generation of long-term T-cell memory (155). An increase in CD4 cytotoxic T cells was found in a mouse pancreatic cancer model after the administration of an antigen-specific dendritic cell (DC)-targeted vaccine, and this effect was enhanced when combined with anti-CTLA4 therapy (156). Antitumor activity mediated by cytotoxic CD4 T cells was also shown in a model of hepatocellular carcinoma treated with a DC vaccine and interleukin-12 (IL-12) (157). Finally, we recently reported that CD4 T cells specific for the cancer-testis antigen NY-ESO-1, either naturally occurring or induced by long synthetic peptide immunization in combination with CpG (158), were able to efficiently kill tumour cells in an MHC class II-restricted manner (73).

## 6 Conclusions

Our understanding of the phenotypic and functional heterogeneity of CD4 T cells has progressed enormously from the 1990s, when multicolour flow cytometry and cytokine release assays were the main tools at hand, to the last seven years or so with the advent of single-cell-resolution technologies. These include mass cytometry and single-cell RNA sequencing. In addition to the firm establishment of broadly defined type 1, 2 and 3 CD4 T-cell functional subsets, the wealth of available results supports the inclusion of an additional specialized functional subset uniquely able to kill target cells in an MHC-II-restricted antigen-specific manner. Unlike types 1 to 3, which can be defined by specific cell clients (phagocytes for type 1, B- cells, eosinophils and mast cells for type 2 and stromal and epithelial cells for type 3), the cytolytic subset targets all cell types and tissues.

A major caveat to the breadth of CD4 CTL responsiveness is the restricted tissue expression of MHC-II molecules. Indeed, their expression is known to be confined to antigen-presenting cells and phagocytes. However, this pattern only holds true in steady state tissue conditions. In fact, MHC class II expression is inducible by IFN-γ, a cytokine produced during ongoing adaptive immune responses mediated by both CD4 T-cell and MHC-I restricted CD8 T-cell responses. It is thus conceivable that CD4 CTLs are an important component of adaptive immunity poised to be deployed during acute as well as sustained immunity.

As with practically every cell-mediated immune response, CD4 CTLs may subserve potent protective immune responses against microbial pathogens as well as cancer and contribute to inflammatory and autoimmune diseases. The signals and regulatory gene expression regulators involved in the specification of CD4 CTL lineage commitment and differentiation are understood. However, much work remains to be done to conclusively outline the mechanisms involved, the stability of the lineage and the regulation of its induction and maintenance. The evidence supporting the importance of CD4 CTLs in health, longevity and immunity provides impetus for these future studies. A detailed understanding of CD4 CTLs may enrich the armamentarium of the blooming field of immunotherapy.

## Author Contributions

MC and MS drafted the manuscript. PR and CJ critically revised the manuscript. All authors contributed to the article and approved the submitted version.

## Funding

This work was funded by grants from the Swiss National Foundation (PRIMA PR00P3_179727 to CJ, 310030_182735 to PR), the Fondazione San Salvatore (to CJ). MC is supported by an iGE3 PhD salary award. This study received funding from the Société Académique Vaudoise supported by the Novartis Consumer Health. The funder was not involved in the study design, collection, analysis, interpretation of data, the writing of this article or the decision to submit it to publication. The authors declare no other competing interests.

## Conflict of Interest

The authors declare that the research was conducted in the absence of any commercial or financial relationships that could be construed as a potential conflict of interest.

## Publisher’s Note

All claims expressed in this article are solely those of the authors and do not necessarily represent those of their affiliated organizations, or those of the publisher, the editors and the reviewers. Any product that may be evaluated in this article, or claim that may be made by its manufacturer, is not guaranteed or endorsed by the publisher.
